# Climate-Resilient F_3_ Progenies of *Coffea arabica*: Agronomic Traits and Antibiosis to *Hypothenemus hampei*

**DOI:** 10.3390/plants14243744

**Published:** 2025-12-09

**Authors:** Diana Molina, Claudia Patricia Flórez-Ramos, Esther Cecilia Montoya, Rubén Medina, Pablo Benavides

**Affiliations:** 1 Plant Breeding Department, National Coffee Research Center (Cenicafé), Manizales 170009, Colombia; claudia.florez@cafedecolombia.com; 2 Statistics Department, National Coffee Research Center (Cenicafé), Manizales 170009, Colombia; esthercecilia.montoya@cafedecolombia.com (E.C.M.); ruben.medina@cafedecolombia.com (R.M.); 3 Entomology Department, National Coffee Research Center (Cenicafé), Manizales 170009, Colombia; pablo.benavides@cafedecolombia.com

**Keywords:** antibiosis, coffee berry borer, coffee breeding, modeling

## Abstract

Climate change is expected to reduce coffee yields and intensify infestations by *Hypothenemus hampei*, the most destructive coffee pest worldwide. Strengthening host plant resistance offers a sustainable approach to mitigate these impacts. This study aimed to characterize F_3_ progenies derived from crosses between Castillo^®^—a variety with high agronomic performance and resistance to *Hemileia vastatrix*—and Ethiopian *Coffea arabica* introductions exhibiting antibiosis to *H. hampei* for agronomic traits and, for the first time, modeled reductions in *H. hampei* infestation under projected climate change scenarios. Thirteen F_3_ progenies with medium plant stature, rust resistance, and high productivity were selected using a 6 × 7 lattice design. Antibiosis was quantified under controlled conditions by infesting individual coffee beans with a single female borer and validated under field conditions by artificially infesting productive branches with 100 females. Relative to the susceptible control, oviposition decreased by 18.0–25.8% under controlled conditions and by 24.1–69.8% in the field. To anticipate progeny performance under warmer conditions, simulation modeling integrating laboratory and field data under Neutral and El Niño scenarios for the Naranjal and Paraguaicito experimental stations, indicated that progenies exhibiting 34–55% reductions in oviposition would maintain infestation below the economic damage threshold (5%) throughout the eight-month fruit development period. Progenies with the highest antibiosis (55%) would reach the action threshold (2%) only in the seventh month. These findings demonstrate the potential of antibiosis-based resistance to reduce insecticide use and strengthen integrated pest management under projected climate change scenarios.

## 1. Introduction

Recent taxonomic revisions indicate that the genus *Coffea* comprises nearly 132 species [1]. Among these, Arabica (*Coffea arabica* L.) and Robusta (*Coffea canephora* Pierre ex A. Froehner) dominate global production and trade, contributing an estimated USD 245.6 billion to the international coffee market [2] and supporting the livelihoods of nearly 25 million growers, most of them smallholders [3,4]. Arabica accounts for 56.6% of global production and is widely recognized for its superior sensory attributes [5]. It is typically cultivated at elevations of 950–1950 m a.s.l. under climatic conditions similar to those of its center of origin [6], with optimal annual mean temperatures of 18–21 °C [7]. In Colombia, Arabica coffee is the most economically significant agricultural product and the second-most traded commodity after oil, with an estimated harvest value of COP 16.1 billion in 2024 [8].

Climate projections indicate that Arabica is highly vulnerable to increases in air temperature of 1.7–2.5 °C and to broader regional warming trends [9,10]. Such changes are expected to shift cultivation to higher elevations, reducing the extent of suitable coffee-growing areas, even within Ethiopia, the species’ center of origin [11,12,13,14,15,16]. Rising temperatures are also anticipated to lower global coffee production [17,18] and increase susceptibility to the coffee berry borer (*Hypothenemus hampei* Ferrari), whose reproductive rate and geographic distribution expand under warmer conditions [12,19,20].

*H. hampei* is the most destructive insect pest of coffee in Colombia and in nearly all coffee-producing countries, except Nepal and Australia [21]. Fertile females bore into berries 120–150 days after flowering, once dry matter exceeds 20% [22], creating tunnels and galleries where they oviposit [23]. Emerging larvae feed on the endosperm, reducing parchment weight [24], and infested berries often exhibit sensory deterioration due to fungal contamination [25,26,27]. Because females remain inside the berry and oviposit over extended periods (7–50 days), all life stages may coexist within a single fruit [28]. Under controlled conditions, the insect completes its life cycle in 28 days at 26 °C in parchment beans with 40% moisture [29], whereas, in the field, at 20.7–21.6 °C, development requires 45–60 days [30].

Integrated pest management (IPM) strategies developed by Cenicafé have successfully maintained *H. hampei* populations below the economic damage threshold [31]. However, projected climate change scenarios characterized by higher temperatures and more frequent and intense El Niño–Southern Oscillation (ENSO) events [32] present new challenges. In Colombia, ENSO events result in reduced rainfall and increased air temperature, solar radiation, and brightness across most coffee-growing regions [33]. These conditions enhance reproductive capacity and shorten the doubling time of *H. hampei* [19,34], particularly in low-elevation and warmer cultivation zones [35].

Host plant resistance (HPR) is a promising complementary IPM strategy. HPR relies on genetically inherited, constitutive plant traits [36] and encompasses three primary mechanisms: antixenosis (reduced host attractiveness), antibiosis (adverse effects on insect development, survival, or fecundity), and tolerance (ability to withstand herbivory without substantial yield loss) [36,37]. HPR has proven highly effective and economically advantageous in many crops [38,39,40], reducing pest damage and production costs while improving yield and quality [38,41]. Despite these advantages, progress in breeding coffee varieties resistant to key pests, including *H. hampei*, has been limited [42,43,44,45], mainly due to scarce resistance sources, long generation times, and the complex nature of resistance mechanisms [41].

Following the introduction of *H. hampei* into Colombia [46], extensive screening of *C. arabica* germplasm identified Ethiopian introductions from Kaffa with pronounced antibiosis effects [29]. These genotypes markedly reduced insect fecundity, resulting in lower net reproductive and intrinsic growth rates and longer population doubling times relative to the susceptible variety Caturra [47]. Importantly, these Ethiopian accessions did not participate in the domestication of *C. arabica* and therefore constitute a valuable genetic reservoir for broadening the species’ narrow genetic base [48,49]. Collected from the humid forests of Kaffa, Illubabor, and Gojjam, west of the Great Rift Valley, these introductions evolved under prolonged geographic isolation, maintained mainly until the nineteenth century due to the Rift Valley barrier and the delayed political integration of Kaffa [50].

Genetic analyses further show that Kaffa accessions cluster within Group I, together with other western Ethiopian introductions, and remain clearly differentiated from eastern accessions [49]. These western introductions possess desirable phenotypic and genetic attributes, including incomplete resistance to *Hemileia vastatrix* Berkeley and Broome [51], resistance to *Meloidogyne incognita* Kofoid and White [52] and *Colletotrichum kahawae* JM Waller and PD Bridge [48], drought tolerance [53], and antibiosis to *H. hampei* [21,42]. Such traits distinguish them from intensively domesticated Arabica cultivars and from eastern Ethiopian accessions, which exhibit more pronounced genetic bottlenecks due to Arabica’s predominantly self-pollinating reproductive system [49]. This erosion of gene diversity likely contributed to the loss of resistance to pests and diseases and reduced tolerance to abiotic stressors, as observed for insect resistance in domesticated *C. arabica* [54].

To introgress antibiosis into commercial germplasm, a conventional breeding program was initiated and advanced through the F_1_ and F_2_ generations. Building on these efforts, the present study aimed to characterize the F_3_ progenies derived from these F_2_ populations for key agronomic traits and, for the first time, to model reductions in *H. hampei* infestation under projected climate change scenarios.

## 2. Results

### 2.1. Agronomic Variables

Across the 36 F_3_ progenies, plant height at 24 months after field establishment ranged from 117.2 to 202.3 cm (Table 1). Thirteen progenies (52, 57, 70, 106, 164, 199, 220, 253, 261, 263, 292, 304, and 340) exhibited shorter mean heights (117.2–140.9 cm; *p* ≤ 0.0001) than the commercial controls, corresponding to reductions of 22.0–45.7 cm relative to Caturra (mean: 162.9 cm) and 20.0–43.7 cm relative to Cenicafé 1 (mean: 160.9 cm) (Table 1). In contrast, progenies 42 and 534 exhibited the tallest mean heights (177.5 and 202.3 cm, respectively), both exceeding those of the commercial controls (*p* ≤ 0.0001). The remaining 21 progenies (144.7 to 175.4 cm) did not differ from the controls (*p* ≥ 0.05) (Table 1).

Cumulative yield over three consecutive harvests ranged from 5.9 to 13.0 kg of cherry coffee plant^−1^ (Table 1). Fourteen progenies (15, 21, 304, 311, 324, 363, 371, 373, 391, 406, 416, 489, 534, and 699) produced higher yields (*p* ≤ 0.0001) than Caturra (mean: 6.7 kg plant^−1^) and Cenicafé 1 (mean: 8.0 kg plant^−1^), with gains of 3.9–6.3 kg and 2.6–5.0 kg plant^−1^, respectively. Six additional progenies (42, 128, 220, 297, 354, and 452) yielded 9.3–10.3 kg plant^−1^, exceeding Caturra. The remaining 16 progenies produced yields comparable to those of the commercial controls (*p* ≥ 0.05).

Percentile analysis of *H. vastatrix* incidence revealed that fifteen F_3_ progenies (21, 42, 46, 53, 57, 70, 106, 263, 363, 371, 452, 489, 534, 698, and 699) were resistant, with 70–97% of plants scoring ≤ 3, on the disease-incidence scale [55]. The remaining twenty-one progenies were susceptible, with only 3–67% of plants scoring ≤ 3 (Table 1).

### 2.2. Antibiosis Assessment Under Controlled Conditions

Table 2 summarizes the mean total number of *H. hampei* developmental stages for the thirteen rust-resistant F_3_ progenies (70–97% of plants scoring ≤ 3) and their controls across one to six evaluation periods. In all cases, the F_3_ progenies exhibited fewer developmental stages than the susceptible control, with reductions of 18.0–25.8% and an overall mean reduction of 20.5%.

When group F_3_ progenies and controls were compared across evaluation periods, the contrast test (Fc = 210.6; *p* < 0.0001) indicated fewer developmental stages per bean in the progenies, with >95% confidence, which corresponds to an estimated 19.2% reduction in oviposition (Table 2).

### 2.3. Antibiosis Assessment Under Field Conditions

Across evaluation periods 1–5, progenies consistently exhibited fewer *H. hampei* developmental stages than their susceptible controls (Table 3). Reductions ranged from 24.1% to 69.8%, with an overall mean reduction of 42.4%.

The contrast test for the group F3 progenies (Fc = 383.16; *p* < 0.0001) further confirmed differences in favor of the progenies, with >97% confidence, corresponding to a 35.2% reduction in oviposition (Table 3).

Agronomic and antibiosis traits under controlled and field conditions for the thirteen selected progenies chosen for rust resistance, medium stature, high productivity, and antibiosis are presented in Table 4.

### 2.4. Impact of the Reduction in *H. hampei* Developmental Stages in F_3_ Progenies on the Population Dynamics of the Pest

**Figure 1:**
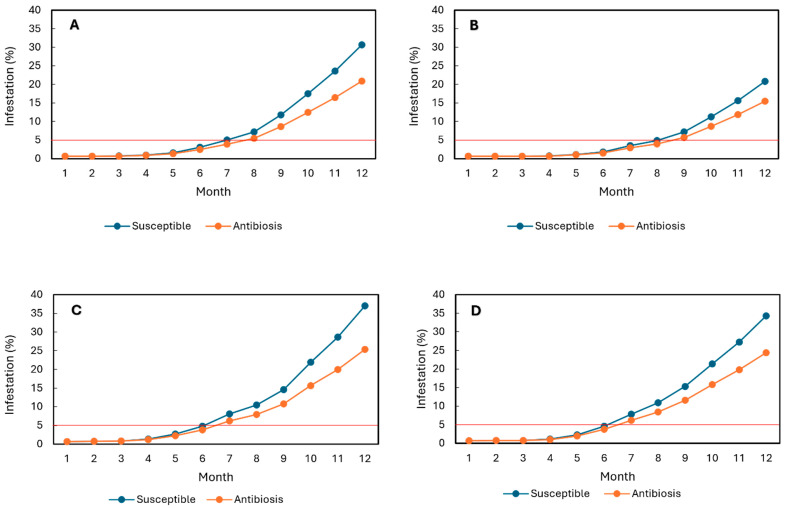
Simulation of *Hypothenemus hampei* infestation in F_3_ progenies exhibiting antibiosis (19% average reduction in total developmental stages under controlled conditions) and in the susceptible Caturra variety over a 12-month fruit development period. (**A**,**B**) Naranjal Experimental Station under El Niño and Neutral conditions, respectively. (**C**,**D**) Paraguaicito Experimental Station under El Niño and Neutral conditions, respectively. The red line indicates the economic damage threshold.

At the Naranjal Experimental Station, simulations indicated that progenies exhibiting an average 19% reduction in developmental stages under controlled conditions would reach the economic damage threshold (5% infestation) after seven months during El Niño and after eight months under Neutral conditions (Figure 1A,B). In contrast, the susceptible variety Caturra reached the economic threshold within seven months under El Niño and within eight months under Neutral conditions (Figure 1A,B).

At the Paraguaicito Experimental Station, progenies exhibiting a 19% reduction in oviposition were predicted to reach 5% infestation between the sixth and seventh months under both climatic scenarios (Figure 1C,D), approximately one month earlier than at Naranjal under El Niño conditions. Caturra, however, reached the economic threshold within six months under both scenarios (Figure 1C,D). These results indicate that a 19% reduction in developmental stages is insufficient to maintain infestation below the economic threshold throughout the entire eight-month fruit development period at either station during El Niño events.

**Figure 2:**
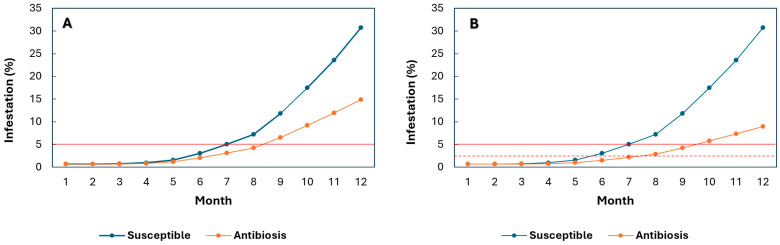
Simulation of *Hypothenemus hampei* infestation during El Niño events in F_3_ progenies exhibiting antibiosis under field conditions, and in the susceptible Caturra variety over a 12-month fruit development period. (**A**) At the Paraguaicito Experimental Station, F_3_ progenies showed an average reduction of 34% in the total number of developmental stages. (**B**) At the Paraguaicito Experimental Station, F_3_ progenies showed an average decrease of 55% in the total number of developmental stages. The solid red line represents the economic damage threshold, and the dashed red line indicates the action threshold (2%).

Because reductions in total developmental stages were substantially greater under field than laboratory conditions, simulations were also conducted using field-based reduction rates. Assuming 34–55% decreases in total developmental stages (Figure 2A,B), progenies at Paraguaicito during El Niño events were predicted to maintain infestation below the economic threshold for 8–9 months. In contrast, Caturra exceeded the threshold 1–2.5 months earlier at 34% and 55% antibiosis, respectively. For progenies exhibiting a 55% reduction, the action threshold (2%) was reached only after the seventh month, approximately one month before the main harvest (Figure 2B). Conversely, in Caturra, the action threshold was reached between the fifth and sixth months (Figure 2B).

## 3. Discussion

Our results demonstrate that antibiosis was inherited in thirteen F_3_ progenies resistant to *H. vastatrix*, with 70–97% of plants scoring ≤ 3 on the incidence scale. These progenies reduced *H. hampei* reproductive fitness by 18.0–25.8% under controlled conditions (Table 2) and by 24.1–69.8% under field conditions (Table 3). Similarly, F_2_ plants previously reduced the total number of *H. hampei* developmental stages (eggs, larvae, prepupae, pupae, and adults) by 18.7–37.7% in laboratory assays and by 29.1–73.1% in the field [42]. Together, these findings confirm that selection based on antibiosis expressed under controlled conditions reliably predicts field performance, facilitating the identification of genotypes with reduced oviposition. The more substantial reductions observed under field conditions likely reflect insect exposure to fluctuating temperatures, which affect development, survival, and fecundity [56]. In contrast, constant laboratory conditions allow *H. hampei* to express its full reproductive potential. This study further demonstrates that antibiosis detected under controlled conditions is maintained and even enhanced in natural field environments due to stronger expression of inducible plant defense mechanisms.

Antibiosis in Ethiopian accessions has been linked to seed proteins, including protease inhibitors (PIs), some of which also function as storage proteins [57]. These compounds inhibit digestive enzymes, reducing amino acid availability for vitellogenesis and thereby decreasing oviposition while negatively affecting development and survival across life stages [58,59]. Antibiosis has been successfully incorporated into coffee breeding, as exemplified by the commercial hybrid Siriema AS1 (*C. arabica × Coffea racemosa*), which reduces larval survival of *Leucoptera coffeella* (Guerin-Meneville and Perrottet) and decreases leaf damage [45]. Similarly, 29 *C. arabica × C. racemosa* progenies expressed antibiosis to *L. coffeella* via reduced larval hatching [60], and antibiosis-based resistance has also been documented in *C. canephora* against *Oligonychus ilicis* McGregor [44].

Developing a composite variety composed of multiple progenies that combine antibiosis to *H. hampei*, resistance to *H. vastatrix*, medium stature, and high yield (Table 4) aligns with the genetic-diversity strategy of Cenicafé’s Coffee Breeding Program. This strategy led to the release of the Colombia variety more than four decades ago [61] and the Castillo^®^ variety, which has been widely cultivated for over twenty years [62], both characterized by durable resistance to *H. vastatrix*. Similarly, the Ruiru 11 variety, whose rust resistance to *H. vastatrix* originated from eight F_4_ progenies derived from the ‘Caturra × Hybrid of Timor’ cross developed by Cenicafé [63], is among the most rust-resistant cultivars in the global coffee-trial network [64].

Selecting medium-statured F_3_ progenies enables higher planting densities and increased productivity per unit area. Five progenies (21, 363, 371, 489, and 699) yielded more than Caturra and Cenicafé 1 (*p* ≤ 0.0001), while progeny 452 yielded more than Caturra alone (Table 1). The remaining seven progenies produced yields comparable to commercial controls, confirming the high productivity of these 13 F_3_ progenies.

Modeling *H. hampei* population dynamics under field-derived antibiosis levels (34–55% reductions in developmental stages) indicated that, during El Niño events in Paraguaicito, total infestation would remain below the economic damage threshold (5%) throughout the eight-month fruit development period (Figure 2A,B). This trait would help maintain coffee production and prevent price penalties associated with deteriorated bean quality when infestation exceeds 5%. Progenies exhibiting the highest antibiosis (55%) would reach the action threshold (2%) only in the seventh month (Figure 2B), allowing harvest with minimal or no insecticide use. Reducing pesticide dependence enhances sustainability by lowering production costs and minimizing agrochemical inputs.

In contrast, susceptible commercial varieties with higher reproductive rates require insecticide applications 1–1.5 months earlier than antibiosis-bearing progenies (Figure 2), once infestation surpasses 2% and more than 50% of females penetrate fruits at positions A and B [31]. At this stage, fruits reach physiological maturity. They are fully susceptible to attack [65], resulting in reduced harvested weight and lower cherry-to-parchment conversion ratios from 5:1 to 8:1, requiring a greater quantity of cherry coffee to obtain 1 kg of dry parchment coffee [66]. Consequently, losses in yield and parchment weight range from 10.82% to 45.12% [24], accompanied by declines in market price as infestation increases [67], and sensory defects caused by microorganisms in bored beans [26,27].

Climatic records show that during strong El Niño events, maximum annual temperatures in Paraguaicito may rise by up to 1.6 °C [68]. Additionally, uneven rainfall distribution produces two pronounced drought periods: one between January and February, affecting the May harvest, and another between June and August (3.5–4.5 months after flowering), which can severely compromise fruit filling during the main November harvest. This period coincides with rapid fruit growth and the critical infestation window, which begins at ~20% dry matter content [65,69]. Under these conditions, progenies that exhibit antibiosis to *H. hampei* and high agronomic performance could help mitigate climate change–driven losses in both yield and bean quality, as demonstrated by the simulations conducted in this study (Figure 2). In contrast, climatic records from Naranjal indicate that during El Niño events, temperature increases (1.2 °C) and rainfall reductions (25%) are less pronounced than those recorded in Paraguaicito (31%) [68]. Moreover, the more evenly distributed rainfall at Naranjal supports fruit filling and stabilizes production [70], suggesting that this site may be less vulnerable to climate-related stress. These contrasting climatic patterns underscore the substantial environmental heterogeneity across Colombian coffee-growing regions and the variable impacts of rising temperatures on *H. hampei* infestation pressure.

Climatic data since 1950 indicate that the Colombian coffee region maintains mean annual temperatures of 18–21 °C, high cloud cover, and abundant rainfall exceeding 2000 mm in most years [68]. These conditions promote multiple flowering events, generating fruits at different developmental stages throughout the year [71] and providing a continuous food supply for *H. hampei*. Rising temperatures associated with climate change and El Niño–driven climatic variability are expected to increase both the number of individuals per generation and the annual number of generations, thereby complicating pest management, particularly at lower elevations.

These projections are consistent with observations from Aguadas (Caldas, Colombia), where lower elevations (<1500 m a.s.l.) exhibit higher infestation levels than higher elevations (>1700 m a.s.l.) due to warmer local temperatures [72]. Similar patterns have been documented in Hawaii, where farms below 1000 m a.s.l. show increased numbers of individuals per fruit and more generations per season at ~200–300 m, attributable to shorter developmental times at elevated temperatures compared with farms at 600–780 m [34]. In eastern Africa, increases in *H. hampei* generations have likewise been predicted for *C. arabica* across elevations of 900–1800 m a.s.l. [20], driven by an 8.5% rise in maximum intrinsic growth rate per 1 °C increase in temperature up to the optimal developmental threshold (26.7 °C), along with reduced developmental time across life stages [19].

Advancing the antibiosis-carrying F_3_ progenies to later generations (F_4_ and F_5_) and evaluating them in low-elevation coffee-growing regions where vulnerability to *H. hampei* is most significant due to higher temperatures [35,41] represents a sustainable strategy to strengthen coffee production in areas where this pest has historically constrained yields. Such progenies may also help compensate for the loss of climatic suitability for coffee cultivation in regions increasingly affected by climate change and escalating infestation pressure. A cultivar that consistently reduces the reproductive fitness of *H. hampei* will lower the number of individuals produced per generation, thereby maintaining pest populations below the economic damage threshold. This approach is economically viable, easily adoptable by growers, and compatible with existing integrated pest management strategies.

## 4. Materials and Methods

### 4.1. Experimental Conditions, Plant Material, and Experimental Design

The study was conducted at the Naranjal Experimental Station of Cenicafé, located in Chinchiná, Caldas, Colombia, on the eastern slope of the Central Andes (4°58′57″ N, 75°36′13″ W). The site is situated at 1381 m a.s.l. and is characterized by a mean annual temperature of 21.4 °C, yearly precipitation of 2782 mm, and an average relative humidity of 77.5%.

The female parents consisted of five Castillo^®^ progenies (CX.2710, CX.2178, CX.2848, CX.2391, and CU.1812), which exhibit desirable agronomic attributes, including intermediate plant stature, resistance to *H. vastatrix*, the most damaging disease affecting coffee production in Colombia and worldwide [73], and high yield potential [63]. The male parents comprised three Ethiopian *Coffea arabica* introductions (CCC.534, CCC.477, and CCC.470) characterized by tall stature, low yield, and the antibiosis trait, expressed as reduced oviposition and fewer *H. hampei* developmental stages relative to the susceptible variety Caturra [29]. The *H. hampei* females used for artificial infestations in laboratory and field assays were obtained from mass-rearing colonies maintained by Biocafé.

A total of 36 third filial generation (F_3_) progenies were evaluated for agronomic performance and antibiosis. These progenies were selected from F_2_ populations described by Molina et al. (2022) [42]. Four female parents and two commercial controls (Caturra and Cenicafé 1) were included, totaling 42 treatments. Genotypes were planted in June 2019 in a 2706 m^2^ plot at a density of 6666 plants ha^−1^ (1.5 m between rows × 1.0 m between plants), following a 6 × 7 lattice design. Each experimental unit consisted of a 12-plant row, with 10 effective plants per plot and three replicates per treatment, for a total of 30 plants. Ethiopian male introductions were excluded from field planting due to excessive plant height.

Seedlings were produced by germinating 100 seeds per treatment. From these, 50 normal, vigorous plants with 2–4 pairs of true leaves were transplanted into 17 × 23 cm polyethylene bags containing 2.2 kg of soil. Abnormally developed seedlings were discarded. Fertilization and soil acidity correction were applied according to soil test recommendations.

At 24 months after field establishment, plant height (measured from the soil base to the apex) and cumulative yield across three consecutive harvests (2021–2023), expressed as kilograms of cherry coffee plant^−1^, were recorded. *H. vastatrix* incidence was evaluated in April and August of 2021, 2022, and 2023 using Scale I of Eskes and Toma-Braghini (1981) [54]. Chemical control was applied only to the susceptible control Caturra.

For the laboratory antibiosis evaluation, mature fruits (200–240 days old) were collected weekly and processed as described by Molina et al. (2022) [42]. Healthy parchment beans (40% moisture) were placed individually in borosilicate vials and infested with a single fertile *H. hampei* female. Each vial was sealed with a perforated plastic lid (1 mm opening). A total of 2400 experimental units per treatment (80 per plant) were established in a completely randomized design. At 28 days post-infestation, beans were dissected to quantify eggs, larvae, prepupae, pupae, and adults under a stereomicroscope (ZEISS Stemi; Carl Zeiss Microscopy GmbH, Oberkochen, Germany). A minimum of 29 valid units per treatment was required.

Progenies with plant height equal to or shorter than the commercial controls and yield equal to or greater than the controls were selected. Using *H. vastatrix* incidence as a selection criterion, progenies with ≥70% of plants scoring ≤ 3 were retained. From the laboratory antibiosis data, mean total developmental stages per bean and percentage reductions relative to controls were calculated.

To validate antibiosis under field conditions, the same progenies were evaluated using the method of Molina et al. (2022) [42]. Fifty healthy fruits (~150 days old) per plant were selected from three randomly chosen branches and enclosed in entomological sleeves, for a total of 150 fruits. The fruits were infested with 100 *H. hampei* females for 36 h. At 45 days post-infestation, fruits were dissected to quantify all developmental stages; uninfested fruits were discarded.

### 4.2. Statistical Analysis

For agronomic variables, means and standard errors were estimated, and ANOVA was performed using a 6 × 7 lattice design. The least significant difference (LSD) test at the 5% level was applied to identify progenies with performance equal to or superior to the commercial controls. For *H. vastatrix* incidence, the maximum score observed per treatment was recorded, and progenies with ≥70% of plants scoring ≤3 were selected.

For laboratory antibiosis, Duncan’s multiple-range test (*p* ≤ 0.05) was used to compare progenies sharing a common susceptible control. When a progeny had its own control, the LSD test (*p* ≤ 0.05) was applied. For progenies with significantly reduced developmental stages, the percentage reduction relative to the control was calculated descriptively. For field antibiosis, the same procedures were used.

To evaluate the impact of reduced developmental stages on population dynamics, the simulation model of Montoya et al. (2022) [74] was applied to two contrasting sites: Naranjal (1381 m a.s.l.; 2805 mm rainfall; 20.9 °C mean temperature; 1690 sunshine hours year^−1^) and Paraguaicito (1203 m a.s.l.; 2169 mm rainfall; 21.7 °C mean temperature; 1671 sunshine hours year^−1^) [68]. Neutral and El Niño ENSO phases were simulated for each site, and two developmental-stage reduction scenarios were evaluated for each site-ENSO combination.

Initial model inputs included eight infested fruits per tree remaining after harvest; five infested fruits per tree on the ground; two live adults per fruit on the tree; two live adults per fruit on the ground; and an average oviposition rate of three eggs per female per day. Statistical analyses were performed using SAS version 9.4 (SAS Institute Inc., Cary, NC, USA).

## 5. Conclusions

This study achieved substantial advances in the development and evaluation of F_3_ progenies that combine antibiosis against *H. hampei*, resistance to *H. vastatrix*, medium plant stature, and high productivity. Through conventional breeding, the antibiosis trait from Ethiopian *C. arabica* introductions was successfully introgressed into the commercial Castillo^®^ genetic background. Modeling results further indicate that F_3_ progenies expressing 34–55% reductions in oviposition may maintain *H. hampei* infestation below the economic damage threshold throughout the fruit development period, even under El Niño conditions. Progenies exhibiting the highest level of antibiosis (55%) are expected to remain below the action threshold for more than seven months, substantially reducing the need for insecticide applications. These findings underscore the potential of antibiosis-based resistance to provide an effective, sustainable response to the projected impacts of climate change on coffee production.

## Figures and Tables

**Table 1:** Mean values, standard errors (SE) for plant height and yield, and percentage of plants with *Hemileia vastatrix* incidence ≤ 3 on the Eskes and Toma-Braghini scale for 36 F_3_ progenies and the commercial controls.

F_3_ Progenies	N	Height		Production		Rust Percentile
		Mean	EE	Mean	EE	
(CX.2710 × CCC.534)-#21	30	154.3	2.5	10.7 *	0.8	73
(CX.2710 × CCC.534)-#42	30	177.5 **	6.1	9.5	0.4	77
(CX.2710 × CCC.534)-#46	30	156.5	6.6	8.6	0.4	87
(CX.2710 × CCC.534)-#53	29	154.3	5.0	8.7	0.4	97
(CX.2710 × CCC.534)-#57	30	135.0 *	3.4	6.3	0.5	93
(CX.2710 × CCC.534)-#70	29	138.9 *	7.5	7.6	0.5	93
(CX.2710 × CCC.534)-#106	28	123.6 *	5.4	8.5	1.2	93
(CX.2178 × CCC.470)-#263	27	131.6 *	5.6	7.2	2.3	70
(CX.2848 × CCC.477)-#363	30	158.4	6.4	10.6 *	0.6	87
(CX.2848 × CCC.477)-#371	30	163.8	4.6	11.3 *	1.0	70
(CX.2391 × CCC.477)-#452	30	144.7	3.9	10.3	0.4	73
(CX.2391 × CCC.477)-#489	30	161.8	5.8	10.9 *	1.4	87
(CU.1812 × CCC.534)-#698	29	154.8	6.6	8.2	0.4	70
(CU.1812 × CCC.534)-#699	29	167.1	2.9	11.1 *	0.5	77
(CX.2391 × CCC.477)-#534	30	202.3 **	2.8	10.8 *	0.5	70
(CX.2710 × CCC.534)-#15	30	164.3	4.7	11.3 *	0.2	43
(CX.2710 × CCC.534)-#52	26	129.6 *	4.9	8.6	2.0	67
(CX.2710 × CCC.534)-#128	29	147.9	6.3	9.8	0.6	67
(CX.2178 × CCC.470)-#177	28	165.7	6.6	8.5	1.0	53
(CX.2178 × CCC.470)-#164	25	140.4 *	8.1	6.9	0.6	67
(CX.2178 × CCC.470)-#199	26	130.0 *	3.3	8.8	1.0	57
(CX.2178 × CCC.470)-#220	30	119.7 *	3.5	9.5	1.4	65
(CX.2178 × CCC.470)-#253	30	117.2 *	4.4	5.9	0.6	33
(CX.2178 × CCC.470)-#261	27	125.8 *	6.4	6.8	1.5	50
(CX.2178 × CCC.470)-#292	28	140.8 *	6.1	7.6	1.3	33
(CX.2178 × CCC.470)-#297	28	147.1	6.6	9.9	1.×	17
(CX.2848 × CCC.477)-#304	30	140.9 *	3.8	11.4 *	1 × 9	3
(CX.2848 × CCC.477)-#311	30	164.5	5.1	11.5 *	0.×	10
(CX.2848 × CCC.477)-#313	29	153.5	6.5	8.2	0.×	37
(CX.2848 × CCC.477)-#324	29	162.3	2.2	12.3 *	0.×	10
(CX.2848 × CCC.477)-#340	29	137.9 *	3.9	7.9	0.×	40
(CX.2848 × CCC.477)-#354	30	149.2	5.4	9.3	0.×	17
(CX.2848 × CCC.477)-#373	29	173.3	6.3	13.0 *	0.×	30
(CX.2848 × CCC.477)-#391	30	145.5	3.5	11.9 *	0 × 5	7
(CX.2848 × CCC.477)-#406	30	171.1	4.3	12.2 *	0.×	33
(CX.2848 × CCC.477)-#416	20	175.4	5.2	11.4 *	0.4	45
Caturra	30	162.9	2.6	6.7	0.6	0
Cenicafé 1	29	160.9	3.1	8.0	0.7	30
CX.2178	30	169.7	2.6	10.6	0.6	23
CX.2391	30	161.3	3.1	7.4	0.2	57
CX.2710	30	165.7	3.4	7.5	0.2	80
CX.2848	30	176.2	3.6	6.0	0.7	3

* Height lower than the commercial controls according to the least significant difference (LSD) test at *p* ≤ 0.05. ** Height higher than the commercial controls according to the LSD test at *p* ≤ 0.05. * Yield higher than Cenicafé 1 according to the LSD test at *p* ≤ 0.05.

**Table 2:** Mean values, standard errors (SE), and percentage reduction in *Hypothenemus hampei* developmental stages of F_3_ progenies and their respective susceptible controls under controlled conditions.

F_3_ Progeny	Number Coffee Beans	*H. hampei* Stages			(%) Reduction *H. hampei* Stages
		Mean		SE	
(CX2391 × CCC477)-#489	106	12.4	B ^1^	0.4	22.9
(CX2848 × CCC477)-#363	59	13.1	B ^1^	0.7	18.5
Susceptible control	57	16.1	A	0.7	
(CX2710 × CCC534)-#57	97	16.2	B ^1^	0.6	18.4
(CX2848 × CCC477)-#363	103	14.9	B ^1^	0.5	24.9
Susceptible control	52	19.9	A	0.9	
(CU1812 × CCC534)-#699	60	17.1	B ^1^	0.7	23.3
(CX2178 × CCC470)-#263	56	18.1	B ^1^	1.1	19.1
(CX2710 × CCC534)-#106	58	17.2	B ^1^	0.9	23.0
Susceptible control	50	22.3	A	1.2	
(CU1812 × CCC534)-#698	49	13.2	B ^1^	0.7	21.9
(CX2178 × CCC470)-#263	29	13.8	B ^1^	1.0	18.1
Susceptible control	45	16.9	A	0.8	
(CX2178 × CCC470)-#263	120	17.4	B ^1^	0.7	21.4
(CX2710 × CCC534)-#53	56	17.4	B ^1^	1.1	21.1
Susceptible control	56	22.1	A	1.5	
(CU1812 × CCC534)-#699	59	17.6	B ^1^	0.9	22.2
(CX2391 × CCC477)-#489	60	18.5	B ^1^	0.7	18.7
Susceptible control	50	22.7	A	1.5	
(CX2710 × CCC534)-#106	60	15.4	B ^1^	0.7	24.1
(CX2848 × CCC477)-#363	45	16.6	B ^1^	1.0	18.5
Susceptible control	49	20.3	A	1.2	
(CX2391 × CCC477)-#452	57	18.7	B ^1^	0.7	19.2
(CX2710 × CCC534)-#106	109	18.4	B ^1^	0.6	20.7
(CX2848 × CCC477)-#371	47	18.9	B ^1^	0.9	18.3
Susceptible control	50	23.2	A	1.2	
(CU1812 × CCC534)-#699	51	18.0	B ^1^	0.9	19.4
(CX2178 × CCC470)-#263	39	17.6	B ^1^	1.1	21.1
(CX2710 × CCC534)-#21	47	18.3	B ^1^	1.0	18.1
Susceptible control	47	22.3	A	1.1	
(CX2710 × CCC534)-#53	88	18.0	B ^1^	0.7	21.8
(CX2848 × CCC477)-#363	39	17.1	B ^1^	1.2	25.8
Susceptible control	57	23.0	A	1.1	
(CX2710 × CCC534)-#106	53	21.4	B ^1^	1.2	18.3
(CX2710 × CCC534)-#70	51	21.1	B ^1^	1.2	19.6
(CX2848 × CCC477)-#371	50	21.5	B ^1^	1.0	18.0
Susceptible control	53	26.2	A	1.1	
(CX2178 × CCC470)-#263	44	23.5	B ^1^	1.2	18.9
(CX2710 × CCC534)-#106	57	22.2	B ^1^	1.1	23.5
Susceptible control	55	29.0	A	1.3	
(CX2710 × CCC534)-#70	49	11.8	B ^2^	0.6	21.5
Susceptible control	51	15.1	A	0.8	
(CX2710 × CCC534)-#21	54	15.9	B ^2^	0.7	18.3
Susceptible control	59	19.5	A	0.9	
(CX2391 × CCC477)-#452	48	15.2	B ^2^	0.7	22.3
Susceptible control	49	19.6	A	1.0	
(CU1812 × CCC534)-#698	52	18.9	B ^2^	1.0	18.2
Susceptible control	51	23.1	A	1.2	
(CX2710 × CCC534)-#21	62	17.7	B ^2^	0.9	20.9
Susceptible control	52	22.4	A	1.1	
(CX2391 × CCC477)-#489	66	18.2	B ^2^	0.7	18.5
Susceptible control	50	22.3	A	1.2	
(CX2710 × CCC534)-#46	57	17.8	B ^2^	0.7	18.7
Susceptible control	64	22.0	A	0.9	
(CX2178 × CCC470)-#263	53	13.0	B ^2^	0.6	19.0
Susceptible control	50	16.1	A	1.0	
Group F_3_ Progenies	2190	17.2		0.1	19.2
Susceptible control	1047	21.2		0.3	

^1^ Values followed by different letters differ significantly according to Duncan’s multiple range test at *p* ≤ 0.05. ^2^ Values followed by different letters differ significantly according to the least significant difference test at *p* ≤ 0.05.

**Table 3:** Mean values, standard errors (SE), and percentage reduction in *Hypothenemus hampei* developmental stages of F_3_ progenies and their susceptible controls under field conditions.

Progeny	Number Coffee Beans	*H. hampei* Stages			(%) Reduction *H. hampei* Stages
		Mean		SE	
(CU1812 × CCC534)-#698	79	11.2	ED ^1^	0.5	41.2
(CX2178 × CCC470)-#263	219	12.8	CB ^1^	0.3	32.9
(CX2391 × CCC477)-#489	89	10.3	E ^1^	0.4	45.6
(CX2710 × CCC534)-#21	101	14.2	B ^1^	0.6	25.3
(CX2848 × CCC477)-#363	75	12.5	DC ^1^	0.5	34.0
Susceptible control	43	19.0	A	0.6	
(CU1812 × CCC534)-#699	15	3.4	B ^1^	1.0	69.8
(CX2710 × CCC534)-#106	24	5.2	B ^1^	0.9	53.8
Susceptible control	41	11.3	A	0.9	
(CX2178 × CCC470)-#263	114	11.0	B ^1^	0.5	37.1
(CX2710 × CCC534)-#106	28	9.1	B ^1^	0.6	47.9
(CX2848 × CCC477)-#371	23	9.8	B ^1^	1.0	44.0
Susceptible control	38	17.6	A	1.1	
(CU1812 × CCC534)-#698	58	18.1	B ^1^	1.2	27.1
(CX2178 × CCC470)-#263	16	15.8	B ^1^	1.0	36.5
(CX2391 × CCC477)-#452	59	18.8	B ^1^	1.1	24.1
(CX2391 × CCC477) #489	71	9.2	B ^1^	0.7	63.1
(CX2710 × CCC534)-#53	121	18.6	B ^1^	0.7	25.1
Susceptible control	71	24.8	A	1.6	
(CX2710 × CCC534)-#57	28	9.1	B ^1^	0,8	56.9
(CX2848 × CCC477)-#363	129	10.3	B ^1^	0.6	51.0
Susceptible control	75	21.0	A	1.1	
(CX2710 × CCC534)-#106	49	8.0	B ^1^	0.6	46.7
(CX2848 × CCC477)-#363	65	6.4	B ^1^	0.3	57.7
Susceptible control	51	15.1	A	1.3	
(CX2391 × CCC477)-#489	47	7.0	B ^1^	0.5	52.4
(CX2710 × CCC534) #70	27	8.0	B ^1^	0.6	45.6
(CX2848 × CCC477) #371	20	7.8	B ^1^	0.4	47.3
Susceptible control	36	14.7	A	1.2	
(CU1812 × CCC534) #699	42	14.3	B ^1^	1.1	35.1
(CX2391 × CCC477)-#452	46	13.9	B ^1^	1.0	37.1
(CX2710 × CCC534)-#106	65	12.7	CB ^1^	0.6	42.5
(CX2710 × CCC534)-#21	65	10.7	C ^1^	0.5	51.7
(CX2710 × CCC534)-#46	54	14.7	B ^1^	1.0	33.5
(CX2710 × CCC534)-#57	61	13.8	B ^1^	0.7	37.6
Susceptible control	66	22.1	A	0.6	
(CX2710 × CCC534)-#53	26	12.8	B ^2^	1.1	28.4
TESTIGO	62	17.8	A	1.1	
(CX2710 × CCC534)-#70	25	11.6	B ^2^	0.9	30.0
Susceptible control	56	16.6	A	0.6	
(CX2710 × CCC534)-#106	25	8.0	B ^2^	0.4	55.0
Susceptible control	53	17.8	A	0.9	
Group F3 progenies	1866	12.1		0.1	35.2
Susceptible control	592	18.6		0.4	

^1^ Values followed by different letters differ significantly according to Duncan’s multiple range test at *p* ≤ 0.05. ^2^ Values followed by different letters differ significantly according to the least significant difference test at *p* ≤ 0.05.

**Table 4:** Mean values and standard errors (SE) of agronomic traits and antibiosis parameters, and percentage reduction in *Hypothenemus hampei* developmental stages in F_3_ progenies evaluated under controlled and field conditions.

F_3_ Progeny	Agronomic Variables					Antibiosis Controlled Conditions			Antibiosis Field Conditions		
	Height		Production			# Stages/Bean			# Stages/Fruit		
	Mean	SE	Mean	SE	*H. vastatrix* Incidence	Mean	SE	(%) Reduction *H. hampei* Stages	Mean	SE	(%) Reduction *H. hampei* Stages
(CX.2710 × CCC.534)-#53	154.3	9.8	8.7	0.4	97	17.8	0.6	21.4	17.6	0.6	26.8
(CX.2710 × CCC.534)-#57	135.0	6.7	6.3	0.5	93	16.2	0.6	18.4	12.3	0.6	47.2
(CX.2710 × CCC.534)-#70	138.9	14.7	7.6	0.5	93	16.5	0.8	20.6	9.8	0.6	37.8
(CX.2710 × CCC.534)-#106	123.6	10.6	8.5	1.2	93	18.8	0.4	21.9	9.4	0.4	49.2
(CX.2710× CCC.534)-#46	156.5	12.9	8.6	0.4	87	17.8	0.7	18.7	14.7	1.0	33.5
(CX.2848 × CCC.477)-#363	158.4	12.6	10.6	0.6	87	15.1	0.4	21.9	10.0	0.3	47.6
(CX.2391 × CCC.477)-#489	161.8	11.4	10.9	1.4	87	15.6	0.4	20.0	9.2	0.3	53.7
(CU.1812 × CCC.534)-#699	167.1	5.6	11.1	0.5	77	17.6	0.5	21.6	11.4	1.0	52.5
(CX.2710 × CCC.534)-#21	154.3	4.9	10.7	0.8	73	17.3	0.5	19.1	12.8	0.4	38.5
(CX.2391 × CCC.477)-#452	144.7	7.6	10.3	0.4	73	17.1	0,5	20.7	16.7	0.8	30.6
(CX.2178 × CCC.470)-#263	131.6	10.9	7.2	2.3	70	17.3	0,4	19.6	12.3	0.3	35.3
(CX.2848 × CCC.477)-#371	163.8	9.1	11.3	1.0	70	20.2	0.7	18.1	8.9	0.6	45.6
(CU.1812 × CCC.534)-#698	154.8	12.9	8.2	0.4	70	16.1	0.7	20.1	14.1	0.6	34.1

## Data Availability

The original contributions presented in the study are included in the article; further inquiries can be directed to the corresponding author.
